# Fenofibrate as a PPARα Agonist Modulates Neuroinflammation and Glutamate Receptors in a Rat Model of Temporal Lobe Epilepsy: Region-Specific Effects and Behavioral Outcomes

**DOI:** 10.3390/ijms26189054

**Published:** 2025-09-17

**Authors:** Anna A. Kovalenko, Maria V. Zakharova, Olga E. Zubareva, Alexander P. Schwarz, Yury A. Skorik, Aleksey V. Zaitsev

**Affiliations:** 1 Laboratory of Molecular Mechanisms of Neural Interactions, Sechenov Institute of Evolutionary Physiology and Biochemistry of the Russian Academy of Sciences, 194223 Saint Petersburg, Russia; kovalenko_0911@mail.ru (A.A.K.); zaharova-masha@yandex.ru (M.V.Z.); zubarevaoe@mail.ru (O.E.Z.); aleksandr.pavlovich.schwarz@gmail.com (A.P.S.); yury_skorik@mail.ru (Y.A.S.); 2 Almazov National Medical Research Centre, 197341 Saint Petersburg, Russia; 3 Branch of Petersburg Nuclear Physics Institute Named by B.P. Konstantinov of National Research Centre «Kurchatov Institute»—Institute of Macromolecular Compounds, 199004 Saint Petersburg, Russia

**Keywords:** temporal lobe epilepsy, fenofibrate, PPARα agonist, neuroinflammation, glutamate receptors, region-specific effects, drug repurposing, behavioral comorbidities, latent phase, lithium-pilocarpine model

## Abstract

Temporal lobe epilepsy (TLE) remains pharmacoresistant in 30–40% of patients. Peroxisome proliferator-activated receptor alpha (PPARα) agonists like fenofibrate exhibit anti-inflammatory and neuroprotective properties, but their region-specific effects during epileptogenesis and on behavioral comorbidities are unknown. We investigated fenofibrate (100 mg/kg, 7 days) in the lithium-pilocarpine rat model during the latent phase. Fenofibrate (1) reduced anxiety-like behaviors and improved exploratory deficits; (2) decreased plasma short-chain fatty acids (butyric, pentanoic, hexanoic acids); (3) exerted region-specific modulation of glutamate receptors: restored N-methyl-D-aspartate receptor (NMDAR)/α-amino-3-hydroxy-5-methyl-4-isoxazolepropionic acid receptor (AMPAR) subunit gene expression in temporal cortex but failed to reverse and further exacerbated the downregulation of AMPAR subunits in the dorsal hippocampus; (4) prevented the upregulation of cortical neuroinflammation markers (reduced *Nlrp3*, *Il1rn*); and (5) enhanced the A2 astrocyte marker *Ptx3* in the hippocampus while reducing the M2 microglial marker *Arg1* in the temporal cortex. No effects on astrogliosis (*Gfap*), microgliosis (*Aif1*), or trophic factors (*Bdnf*, *Tgfb1*) were observed. This first comprehensive study demonstrates that fenofibrate differentially modulates neuroinflammation and synaptic plasticity across brain regions during epileptogenesis, providing behavioral benefits but highlighting potential hippocampal drawbacks. Its PPARα-mediated actions support further investigation as a complementary strategy for TLE, pending optimization of dosing/timing to mitigate regional disparities.

## 1. Introduction

Epilepsy affects millions worldwide, representing one of the most common neurological disorders. While antiepileptic drugs effectively control seizures in 60–70% of patients [1], a substantial proportion of temporal lobe epilepsy (TLE) remains pharmacoresistant [2], highlighting the urgent need for novel therapeutic strategies targeting fundamental epileptogenic processes. The continuous search for effective therapies is driven by epilepsy’s multifaceted nature, demanding approaches that address not only seizure control but also concomitant cognitive, psychological, and social comorbidities [3]. Compelling evidence implicates neuroinflammation, driven by heightened activation of astroglial and microglial cells, as a key contributor to epileptogenesis and associated comorbidities [4,5]. Consequently, drugs capable of suppressing pro-inflammatory pathways while enhancing neuroprotective glial functions offer a highly promising therapeutic strategy for epilepsy [6].

Peroxisome proliferator-activated receptors (PPARs), ligand-activated nuclear transcription factors, have emerged as compelling therapeutic targets due to their regulatory roles in cellular energy metabolism, inflammation, and neuroprotection [7]. These receptors comprise three subtypes: PPARα, PPARγ, and PPARβ/δ [8]. PPARs serve as a critical interface between the gut microbiota and central regulatory systems [9], modulating gene expression involved in lipid metabolism, oxidative stress responses, and synaptic plasticity. Consequently, PPARα and PPARγ emerge as promising targets for strategies aimed at ameliorating seizure activity and mitigating neuronal damage in chronic epilepsy [10,11]. Crucially, PPAR activation suppresses inflammatory processes, a pivotal pathophysiological mechanism underlying epilepsy [12]. The anti-inflammatory role of PPARα is well-established, evidenced by exaggerated pro-inflammatory responses in macrophages from PPARα-knockout mice [13].

The neuroprotective effects of PPAR agonists have been demonstrated in chronic models of TLE, such as the lithium-pilocarpine model [14,15]. While the effects of PPARγ agonists have been extensively studied [14,16,17,18], research on PPARα agonists remains limited. Existing studies primarily reveal anticonvulsant properties of PPARα agonists in acute seizure models, such as pentylenetetrazole-induced [19] and nicotine-induced seizures [20]. Critically, the precise molecular mechanisms underlying PPARα agonists potential anti-seizure effects, their influence on chronic epileptogenesis, and crucially, their impact on region-specific neuroinflammation, synaptic dysfunction (particularly glutamate receptors), glial phenotype polarization, and behavioral comorbidities during the latent phase are poorly understood.

Fenofibrate, a clinically approved PPARα agonist primarily prescribed for dyslipidemia [21], exhibits systemic anti-inflammatory effects. Its ability to modulate gut microbiota, reduce markers of systemic inflammation, and improve gut barrier function has been demonstrated in models like high-fat diet [22,23]. Furthermore, central PPARα regulates neuronal activity through modulation of nicotinic acetylcholine receptors [20], suggesting PPARα agonists may influence the excitation/inhibition balance relevant to epilepsy.

Building upon the recognized anti-inflammatory and neuromodulatory properties of PPARα activation [8,24], and acknowledging the critical yet underexplored potential of fenofibrate in chronic epileptogenesis, we postulated that intervention with this PPARα agonist during the latent phase of TLE would confer multifaceted neuroprotective effects. Given the well-established differential vulnerability of hippocampal versus cortical circuits to epileptogenic insults [25], coupled with emerging evidence of regionally heterogeneous glial responses in TLE models [26,27], we hypothesized that fenofibrate actions would manifest with regional heterogeneity. We anticipated that fenofibrate would effectively attenuate neuroinflammatory cascades, promote a shift towards neuroprotective glial phenotypes (such as enhancing A2 astrocyte markers and modulating microglial activation), and restore dysregulated glutamatergic neurotransmission. Consequently, we expected these molecular and cellular improvements to translate into measurable amelioration of behavioral comorbidities, such as anxiety and exploratory deficits.

To rigorously evaluate this hypothesis, we employed the lithium-pilocarpine rat model of TLE and administered fenofibrate (100 mg/kg) for 7 days during the latent phase. This model was chosen for study as it is a well-established and widely used model that recapitulates key features of human TLE, including a latent period followed by spontaneous recurrent seizures, hippocampal sclerosis, and associated behavioral comorbidities [25]. Our comprehensive investigation assessed plasma short-chain fatty acid (SCFA) profiles, quantified anxiety-like behaviors and exploratory activity deficits, and conducted a detailed region-specific analysis (dorsal hippocampus vs. temporal cortex) of molecular markers encompassing glutamatergic signaling (N-methyl-D-aspartate receptor (NMDAR)/α-amino-3-hydroxy-5-methyl-4-isoxazolepropionic acid receptor (AMPAR) subunits, glutamate transporters), glial reactivity and polarization (astrocyte/microglia markers, A1/A2, M1/M2 phenotypes), key neuroinflammatory pathways (NLRP3 inflammasome, IL-1β signaling), and neurotrophic factors (BDNF, FGF2, TGFβ1). This study provides the first integrated assessment of fenofibrate’s impact across these interconnected domains during epileptogenesis, explicitly testing its region-dependent efficacy.

## 2. Results

### 2.1. Fenofibrate Administration Has No Effect on Rat Survival or Body Weight in the Latent Phase of the Lithium-Pilocarpine Model

The first week following pilocarpine-induced status epilepticus is a critical period with a high risk of mortality [25]. To assess the potential toxicity of fenofibrate, we monitored animal survival and body weight dynamics.

**Figure 1:**
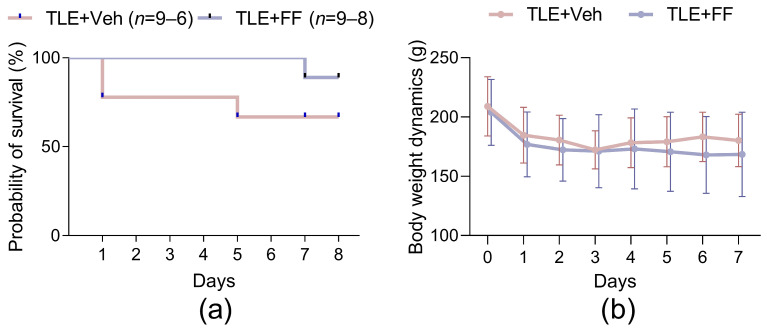
Effect of fenofibrate treatment on survival and body weight dynamics in rats with lithium-pilocarpine model of temporal lobe epilepsy (TLE). (**a**) The survival curves; (**b**) bodyweight dynamics. TLE+Veh (*n* = 9–6), TLE rats; TLE+FF (*n* = 9–8), Fenofibrate-treated TLE rats. Data of body weight are presented as mean ± SD.

Fenofibrate treatment (100 mg/kg, i.p., for 7 days) did not affect the survival rate of rats during the latent phase of the lithium-pilocarpine model. The survival curves for the TLE+Veh and TLE+FF groups were not significantly different (Log-rank test, χ^2^ = 1.4, *p* = 0.234; Figure 1a).

Furthermore, the administration of fenofibrate did not significantly alter the body weight dynamics of the animals. A mixed-design ANOVA revealed a significant effect of time (F_(1.6,22.4)_ = 30.3, *p* < 0.001), reflecting the expected weight change after status epilepticus; however, post hoc analysis did not reveal statistically significant differences. Importantly, there was no significant main effect of treatment (F_(1,16)_ = 0.1, *p* = 0.75) and no significant interaction between time and treatment factors (F_(7,96)_ = 0.99, *p* = 0.437), indicating that fenofibrate did not exacerbate weight loss or influence recovery compared to the vehicle-treated TLE group (Figure 1b).

These results suggest that the chosen regimen of fenofibrate administration is well-tolerated and does not confer additional detrimental effects on the overall survival or physical condition of rats during the latent phase of epileptogenesis.

### 2.2. SCFA Content in Rat Blood Plasma in the Lithium-Pilocarpine Model During Fenofibrate Treatment

**Figure 2:**
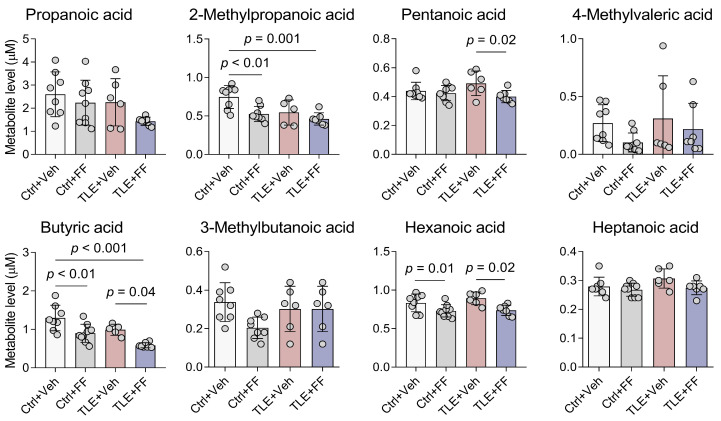
Short-chain fatty acid content in rat plasma during the latent phase of the lithium-pilocarpine model of temporal lobe epilepsy. The analysis was performed on day 8 after seizure induction. Ctrl+Veh (*n* = 7), control rats without treatment; Ctrl+FF (*n* = 9), Fenofibrate-treated Ctrl rats; TLE+Veh (*n* = 6), TLE rats; TLE+FF (*n* = 8), Fenofibrate-treated TLE rats. Two-way ANOVA, only statistically significant results are shown (*p* < 0.05): Butyric acid—F_(1,25)_ (TLE factor) = 12.49, *p* = 0.002, F_(1,25)_ (Treatment factor) = 20.91, *p* < 0.001; 2-Methylpropanoic acid—F_(1,24)_ (TLE factor) = 7.72, *p* = 0.01, F_(1,24)_ (Treatment factor) = 10.19, *p* = 0.004; Pentanoic acid—F_(1,26)_ (Treatment factor) = 5.74, *p* = 0.02; Hexanoic acid—F_(1,26)_ (Treatment factor) = 15.66, *p* < 0.001. Full results of two-way ANOVA are shown in Table A3. Significant differences between groups according to Sidak post hoc test are indicated in the figure. Data are presented as mean ± SD with individual values (circles).

SCFAs, microbial metabolites that can act as endogenous ligands for PPARs [8], were quantified in plasma to assess the systemic pharmacodynamic effects of fenofibrate. A two-way ANOVA revealed that the development of temporal lobe epilepsy (TLE factor) significantly altered the plasma levels of butyric acid and 2-methylpropanoic acid. Fenofibrate treatment (Treatment factor) also induced a significant alteration in the concentrations of several SCFAs, including butyric acid, 2-methylpropanoic acid, pentanoic acid, and hexanoic acid. For all these acids, the interaction between the factors “TLE” and “Treatment” was not significant, indicating that the effect of fenofibrate was consistent and independent of the disease state. Post hoc analysis confirmed that fenofibrate administration significantly reduced the plasma concentrations of butyric acid, hexanoic acid, and 2-methylpropanoic acid in both control (Ctrl+FF) and epileptic (TLE+FF) animals compared to their respective vehicle-treated groups (Figure 2).

The observed reduction in plasma SCFA levels demonstrates a systemic pharmacodynamic response to fenofibrate, indicating its biological activity at the used dosage. The data also demonstrate that pilocarpine-induced epileptogenesis itself can modulate systemic metabolism of butyric acid and 2-methylpropanoic acid, levels of which were also significantly affected by fenofibrate. However, the reduction in these potential PPAR ligands by fenofibrate argues against the hypothesis that its protective effects are mediated by an increase in their systemic levels.

### 2.3. Fenofibrate Ameliorates Specific Exploratory Deficits and Reduces Anxiety-like Behaviors in the Open Field Test

**Figure 3:**
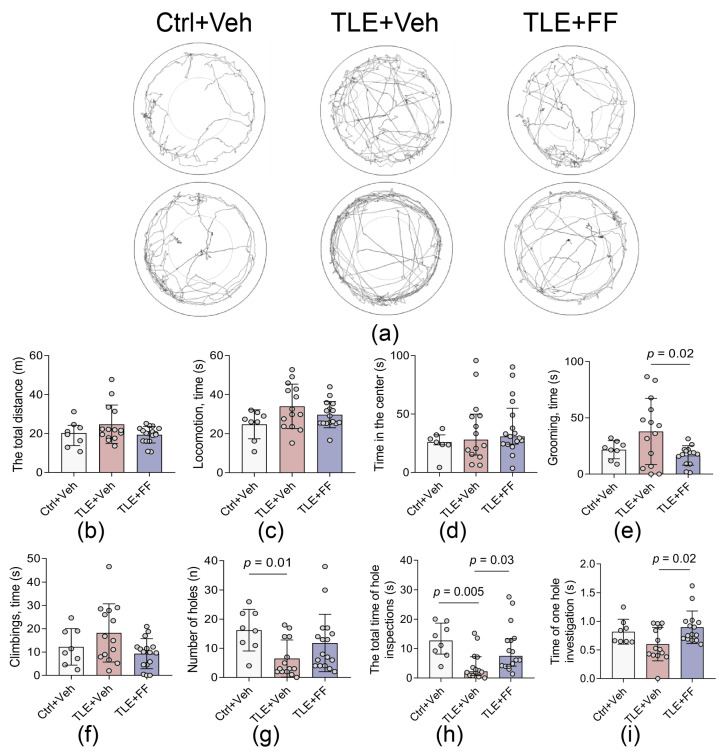
Behavior of control and experimental rats in the Open Field test: (**a**) Examples of tracks. (**b**–**i**) Behavioral patterns. The analysis was performed on day 7 after seizure induction. Ctrl+Veh (*n* = 8), control rats without treatment; TLE+Veh (*n* = 17), TLE rats; TLE+FF (*n* = 17), Fenofibrate-treated TLE rats. One-way ANOVA or Welch ANOVA, or Kruskal–Wallis H test, only statistically significant results are shown (*p* < 0.05): (**e**) Grooming time—F_(2,33)_ = 4.389, *p* = 0.02; (**g**) number of holes—H = 8.38, *p* = 0.015; (**h**) the total time of hole inspections—H = 11.79, *p* = 0.003; (**i**) Time of one hole investigation—F_(2,34)_ = 4.34, *p* = 0.021. Full results of one-way ANOVA or Welch ANOVA, or Kruskal–Wallis H test are shown in Table A3. Significant differences between groups according to Sidak, Games-Howell, or Dunn’s multiple comparisons tests are indicated in the figure. Data are presented as mean ± SD with individual values (circles).

To assess the behavioral comorbidities associated with epileptogenesis, we performed the Open Field test on day 7 after SE (Figure 3). While no significant differences were found in general locomotor activity or anxiety-like behavior, TLE+Veh group exhibited specific deficits in exploratory activity compared to control animals.

A key finding was a significant reduction in the total time spent investigating holes in the TLE+Veh group. Post hoc analysis confirmed this parameter was significantly lower in TLE+Veh rats compared to both Ctrl+Veh and TLE+FF groups (*p* < 0.05, Dunn’s test). The duration of a single hole investigation was also significantly different between groups; however, post hoc analysis reveal significant pairwise differences only between TLE+Veh and TLE+FF groups (*p* < 0.05). The number of holes investigated was significantly lower in TLE+Veh rats compared to Ctrl+Veh (*p* < 0.05, Dunn’s test). The duration of the climbings showed strong variation but did not yield significant difference.

Furthermore, TLE rats displayed a significant increase in self-grooming behavior, which may indicate elevated anxiety-like or compulsive-like activity. Post hoc analysis confirmed that the TLE+FF group showed a significant reduction in grooming time compared to the TLE+Veh group (*p* < 0.05, Sidak’s test).

These results indicate that fenofibrate administration during the latent phase produces a specific behavioral profile, significantly reversing the increase in self-grooming and normalizing the total time spent on exploratory activity, without exerting a generalized effect on locomotor activity or center-field anxiety-like behavior.

### 2.4. Region-Specific Modulation of Ionotropic Glutamate Receptor Gene Expression by Fenofibrate

Numerous studies highlight the critical role of NMDA and AMPA receptors in the pathogenesis of epileptic syndromes [28,29]. Altered expression patterns of NMDA and AMPA receptor subunit genes have been demonstrated both in epilepsy patients and in experimental models of the disease [30,31,32]. As these receptors play a pivotal role in synaptic plasticity [33], disruptions in their subunit composition may not only contribute to seizure generation but also lead to cognitive impairment [34,35]. Given these findings, we firstly examined the expression of genes encoding subunits of ionotropic glutamate NMDA and AMPA receptor subunits in the dorsal hippocampus (DH) and temporal cortex (TC) of rats during the latent phase of the lithium-pilocarpine model of epilepsy.

**Figure 4:**
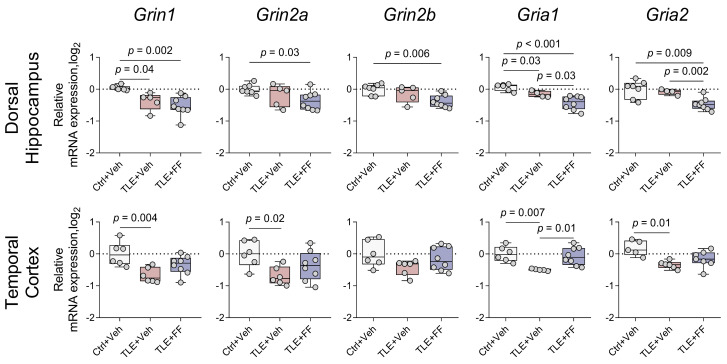
Changes in relative gene expression of NMDA (*Grin1*, *Grin2a*, and *Grin2b*) and AMPA (*Gria1* and *Gria2*) receptor subunits in the dorsal hippocampus (DH) and temporal cortex (TC) of rats. The analysis was performed on day 8 after seizure induction. Ctrl+Veh (*n* = 7), control rats without treatment; TLE+Veh (*n* = 6), TLE rats; TLE+FF (*n* = 8), Fenofibrate-treated TLE rats. One-way ANOVA or Welch ANOVA, only statistically significant results are shown (*p* < 0.05), DH: *Grin1*—F_(2,16)_ = 9.24, *p* = 0.002; *Grin2a*—F_(2,8.7)_ = 4.4, *p* = 0.048; *Grin2b*—F_(2,17)_ = 6.7, *p* = 0.007; *Gria1*—F_(2,9.3)_ = 15.9, *p* = 0.001; *Gria2*—F_(2,16)_ = 9.8, *p* = 0.002; TC: *Grin1*—F_(2,17)_ = 7.2, *p* = 0.006; *Grin2a*—F_(2,17)_ = 4.370, *p* = 0.03; *Gria1*—F_(2,8.1)_ = 19.6, *p* = 0.001; *Gria2*—F_(2,17)_ = 7.952, *p* = 0.004. Full results of one-way ANOVA or Welch ANOVA are shown in Table A3. Significant differences between groups according to Sidak or Games-Howell multiple comparisons tests are indicated in the figure. Data are presented as individual values (circles) with the minimum, the maximum, the sample median, and the first and third quartiles.

Pilocarpine-induced status epilepticus resulted in a widespread downregulation of ionotropic glutamate receptor subunit genes in both the DH and TC during the latent phase (Figure 4).

In the DH, post hoc analysis confirmed significant downregulation in the TLE+Veh group compared to the Ctrl+Veh group for *Grin1* and *Gria1*. Fenofibrate treatment did not reverse these changes. Notably, it exacerbated the downregulation for most subunits. The TLE+FF group showed significantly lower expression than the TLE+Veh group for *Gria1* and *Gria2*. Furthermore, expression levels in the TLE+FF group remained significantly lower than controls for *Grin1*, *Grin2a*, *Grin2b*, and *Gria2*.

In stark contrast, fenofibrate administration in the TC completely prevented the seizure-induced downregulation of key subunits. The mRNA levels of *Grin1*, *Grin2a*, and *Gria1* in the TLE+FF group were restored to control levels and were significantly higher than in the TLE+Veh group (*Gria1*, *p* < 0.01). Although *Gria2* expression in the TLE+FF group was significantly lower than in controls, it was not significantly different from the TLE+Veh group.

**Figure 5:**
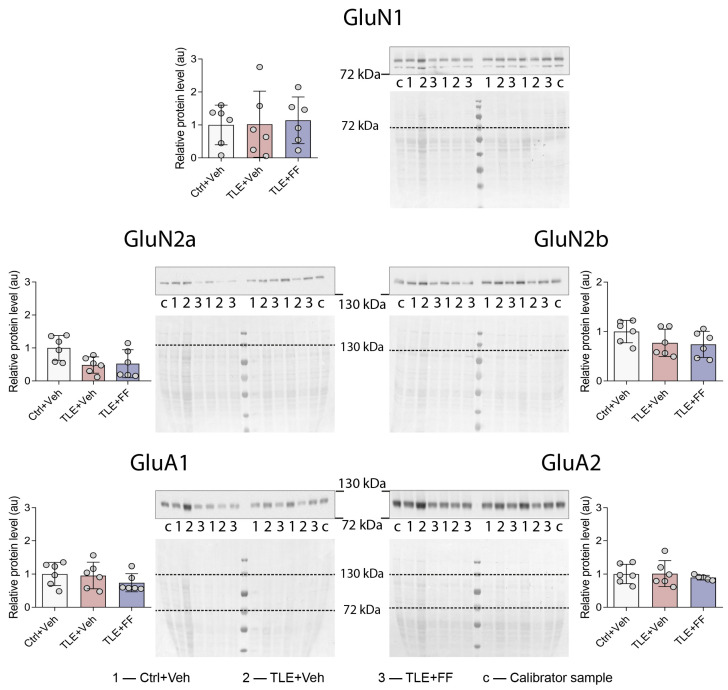
Relative protein levels of NMDA (GluN1, GluN2a, and GluN2b) and AMPA (GluA1 and GluA2) receptor subunits in the cortex of rats. The analysis was performed on day 8 after seizure induction. 1, Ctrl+Veh (*n* = 6), control rats without treatment; 2, TLE+Veh (*n* = 6), 3, TLE rats; TLE+FF (*n* = 6), Fenofibrate-treated TLE rats; c, calibrator sample. Results of statistical tests are shown in Table A3. Data are presented as mean ± SD with individual values (circles).

Despite these profound and opposing transcriptional changes, no significant alterations were found in the protein levels of the corresponding receptor subunits (GluN1, GluN2a, GluN2b, GluA1, GluA2) in the TC (Figure 5). This significant discordance between mRNA and protein levels suggests post-transcriptional regulatory mechanisms may buffer the observed changes in gene expression during the latent period.

### 2.5. Fenofibrate Treatment Results in Decreased Expression of the Neuronal Glutamate Transporter Gene in the Dorsal Hippocampus

The glutamate-glutamine cycle is central to maintaining the balance between excitation and inhibition in the central nervous system [36]. Disruptions in any part of this cycle—whether due to impaired transporter expression or reduced enzymatic activity of glutamine synthetase—can lead to an imbalance in neurotransmission that favors hyperexcitability and the emergence of epileptiform activity [37,38,39]. Synaptic glutamate concentrations are primarily regulated by excitatory amino acid transporters (EAAT1-3), with astrocytic EAAT1 (encoded by *Slc1a3*) and EAAT2 (*Slc1a2*) accounting for ~80% of total glutamate reuptake [40]. EAAT3 (*Slc1a1*) is a neuronal glutamate transporter [41].

**Figure 6:**
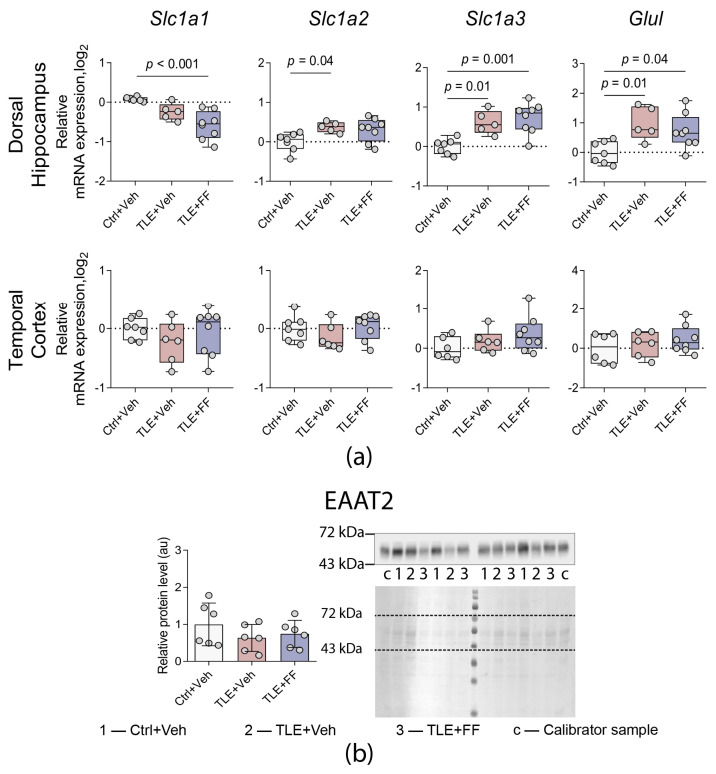
(**a**) Changes in relative gene expression of glutamate transporters *Slc1a1*, *Slc1a2*, *Slc1a3* and glutamine synthetase *Glul* in the dorsal hippocampus and temporal cortex of rats. The analysis was performed on day 8 after seizure induction. Ctrl+Veh (*n* = 7), control rats without treatment; TLE+Veh (*n* = 6), TLE rats; TLE+FF (*n* = 8), Fenofibrate-treated TLE rats. One-way ANOVA, only statistically significant results are shown (*p* < 0.05), DH: *Slc1a1*—F_(2,16)_ = 11.3, *p* = 0.001; *Slc1a2*—F_(2,17)_ = 4.3, *p* = 0.031; *Slc1a3*—F_(2,17)_ = 11.02, *p* = 0.001; *Glul*—F_(2,17)_ = 6.0, *p* = 0.011. Full results of one-way ANOVA are shown in Table A3. Significant differences between groups according to Sidak post hoc test are indicated in the figure. Data are presented as individual values (circles) with the minimum, the maximum, the sample median, and the first and third quartiles. (**b**) Relative protein levels of glutamate transporter EAAT2 in the cortex of rats. The analysis was performed on day 8 after seizure induction. 1, Ctrl+Veh (*n* = 6), control rats without treatment; 2, TLE+Veh (*n* = 6), TLE rats; 3, TLE+FF (*n* = 6), Fenofibrate-treated TLE rats; c, calibrator sample. Data are presented as mean ± SD with individual values (circles).

We analyzed the gene expression of key components of glutamate–glutamine cycle: the astrocytic transporters *Slc1a2* (EAAT2) and *Slc1a3* (EAAT1), the neuronal transporter *Slc1a1* (EAAT3), and glutamine synthetase (*Glul*) (Figure 6a). During the latent phase of the lithium-pilocarpine model, a significant upregulation of astroglial glutamate transporters and glutamine synthetase was observed specifically in the DH. Post hoc analysis confirmed that the expression of *Slc1a2*, *Slc1a3*, and *Glul* was significantly higher in the TLE+Veh group compared to the Ctrl+Veh group (*p* < 0.05 for all comparisons). Fenofibrate administration did not reverse these changes; conversely, it further increased the expression of *Slc1a3* and *Glul*, with the TLE+FF group showing significantly higher mRNA levels than the Ctrl+Veh group (*p* < 0.001 and *p* < 0.05, respectively).

Fenofibrate treatment significantly reduced the expression of the neuronal glutamate transporter gene *Slc1a1* in the DH. Post hoc analysis revealed that mRNA levels in the TLE+FF group were significantly lower than in the Ctrl+Veh group (*p* < 0.001). No significant alterations in the expression of any of these genes were detected in the TC.

The protein level of the major astrocytic glutamate transporter EAAT2 in the TC was also analyzed and found to be unchanged across all groups (Figure 6b), confirming the regional specificity of the observed transcriptional changes.

### 2.6. Administration of Fenofibrate Does Not Affect the Expression of Astroglial and Microglial Marker Genes

**Figure 7:**
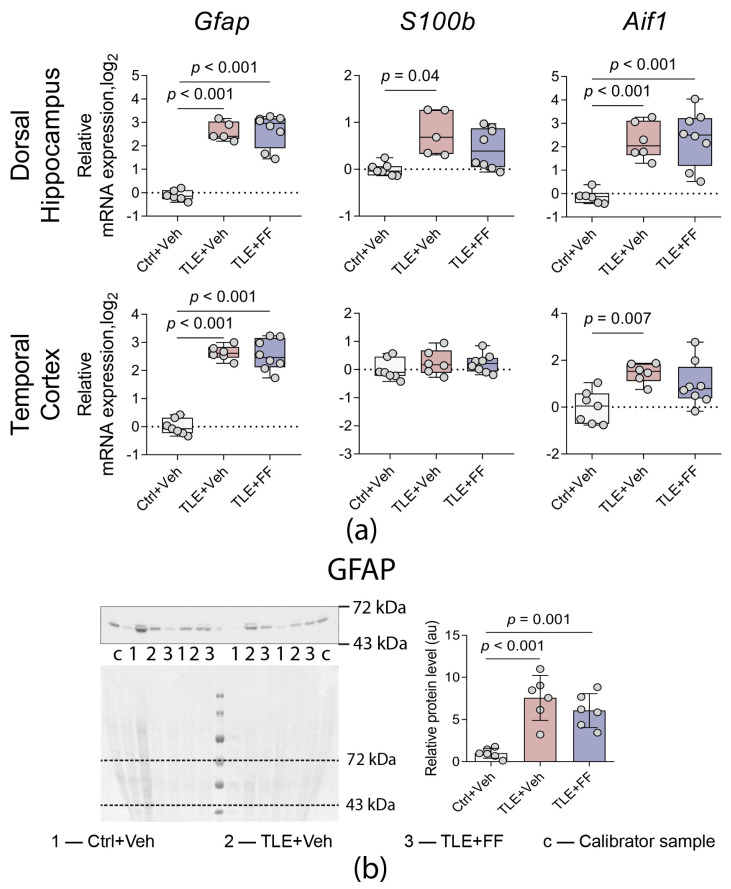
(**a**) Changes in relative gene expression of astrocyte (*Gfap*, *S100b*) and microglial (*Aif1*) markers in the dorsal hippocampus and temporal cortex of rats. The analysis was performed on day 8 after seizure induction. Ctrl+Veh (*n* = 7), control rats without treatment; TLE+Veh (*n* = 6), TLE rats; TLE+FF (*n* = 8), Fenofibrate-treated TLE rats. One-way ANOVA or Welch ANOVA, only statistically significant results are shown (*p* < 0.05), DH: *Gfap*—F_(2,8.9)_ = 118.2, *p* < 0.001; *S100b*—F_(2,7.7)_ = 8.7, *p* = 0.01; *Aif1*—F_(2,17)_ = 15.95, *p* < 0.001; TC: *Gfap*—F_(2,18)_ = 96.4, *p* < 0.001; *Aif1*—F_(2,18)_ = 6.6, *p* = 0.007. Full results of one-way ANOVA or Welch ANOVA are shown in Table A3. Significant differences between groups according to Sidak or Games-Howell multiple comparisons tests are indicated in the figure. Data are presented as individual values (circles) with the minimum, the maximum, the sample median, and the first and third quartiles. (**b**) Changes in relative protein levels of the astrocyte marker GFAP in the cortex of rats. The analysis was performed on day 8 after seizure induction. 1, Ctrl+Veh (*n* = 6), control rats without treatment; 2, TLE+Veh (*n* = 6), TLE rats; 3, TLE+FF (*n* = 6), Fenofibrate-treated TLE rats; c, calibrator sample. One-way ANOVA, F_(2,15)_ = 18.6, *p* < 0.001. Significant differences between groups according to Sidak post hoc test are indicated in the figure. Data are presented as mean ± SD with individual values (circles).

Increased expression of markers for astrocytes (glial fibrillary acidic protein, *Gfap*; S100 calcium-binding protein B, *S100b*) and microglia (allograft inflammatory factor 1, *Aif1*) is a characteristic feature of the latent phase of the lithium-pilocarpine model [42,43,44,45]. We analyzed the expression of these genes in the DH and TC (Figure 7a).

Pilocarpine-induced status epilepticus resulted in a significant increase in the expression of astroglial and microglial markers in both brain regions. In the DH, one-way ANOVA revealed a significant differences on the mRNA levels of *Gfap*, *S100b*, and *Aif1*. Post hoc analysis confirmed that the expression of *Gfap* and *Aif1* was significantly higher in the TLE+Veh group compared to the Ctrl+Veh group (*p* < 0.001 for both). The expression of *S100b* was also significantly elevated in the TLE+Veh group compared to controls (*p* < 0.05).

In the TC, one-way ANOVA also showed a significant alteration on the expression of *Gfap* and *Aif1*. Post hoc tests confirmed that mRNA levels of *Gfap* and *Aif1* were significantly higher in the TLE+Veh group compared to the Ctrl+Veh group (*p* < 0.001 and *p* < 0.01, respectively).

Administration of fenofibrate did not reverse these changes. In the DH, the TLE+FF group exhibited significantly higher expression of *Gfap* and *Aif1* compared to the Ctrl+Veh group (*p* < 0.001 for both), and the expression of *S100b* did not differ significantly from controls. In the TC, the TLE+FF group showed significantly higher *Gfap* mRNA levels compared to the Ctrl+Veh group (*p* < 0.001), while *Aif1* expression did not differ significantly from controls.

Consistent with the transcriptional changes, GFAP protein levels were significantly elevated in the TC of epileptic animals (Figure 7b). Post hoc analysis confirmed that GFAP protein levels were significantly higher in both the TLE+Veh and TLE+FF groups compared to the Ctrl+Veh group (*p* < 0.001 for both comparisons).

These results demonstrate that fenofibrate administration during the latent phase does not significantly attenuate the increased expression of general astroglial and microglial marker genes or GFAP protein levels induced by pilocarpine-induced epileptogenesis.

### 2.7. Fenofibrate Enhances the Expression of Neuroprotective A2 Phenotype Astrocyte Marker but Reduces the Expression of M2 Phenotype Microglia Marker

Reactive astro- and microgliosis are among the most prominent histopathological features of epilepsy [46]. Both astrocytes and microglia can adopt distinct functional states (phenotypes), which determine whether they produce neurotoxic or neuroprotective factors—and thus exert either pro- or anti-epileptogenic effects. For years, a common framework classified glial cells into A1/M1 (pro-inflammatory, neurotoxic) and A2/M2 (anti-inflammatory, neuroprotective) phenotypes [47,48]. While this binary model has been widely used, it is now recognized as overly simplistic, and its applicability remains debated [48,49]. Nevertheless, understanding glial contributions to neuropathologies requires studying the expression of genes associated with both phenotypes [49].

**Figure 8:**
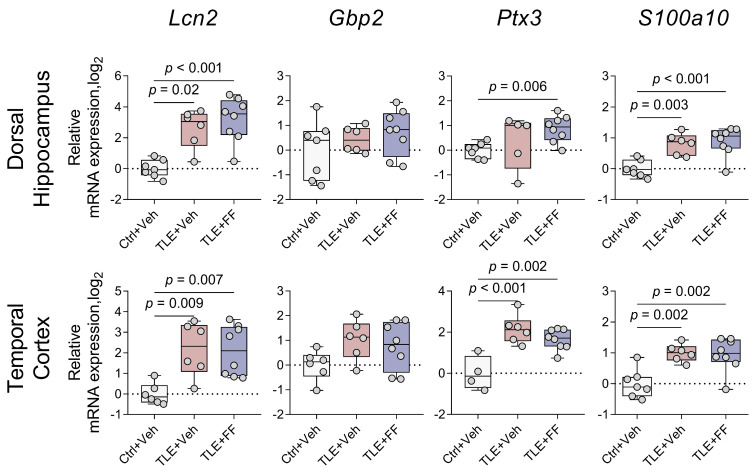
Changes in relative gene expression of astrocyte polarization markers *Lcn2*, *Gbp2* (A1 phenotype) and *Ptx3*, *S100a10* (A2 phenotype) in the dorsal hippocampus and temporal cortex of rats. The analysis was performed on day 8 after seizure induction. Ctrl+Veh (*n* = 7), control rats without treatment; TLE+Veh (*n* = 6), TLE rats; TLE+FF (*n* = 8), Fenofibrate-treated TLE rats. One-way ANOVA or Welch ANOVA, only statistically significant results are shown (*p* < 0.05), DH: *Lcn2*—F_(2,18)_ = 15.2, *p* < 0.001; *Ptx3*—F_(2,8.2)_ = 7.1, *p* = 0.02; *S100a10*—F_(2,18)_ = 12.1, *p* < 0.001; TC: *Lcn2*—F_(2,9.8)_ = 13.6, *p* = 0.001; *Ptx3*—F_(2,15)_ = 13.98, *p* < 0.001; *S100a10*—F_(2,18)_ = 10.5, *p* = 0.001. Full results of one-way ANOVA or Welch ANOVA are shown in Table A3. Significant differences between groups according to Sidak or Games-Howell multiple comparisons tests are indicated in the figure. Data are presented as individual values (circles) with the minimum, the maximum, the sample median, and the first and third quartiles.

**Figure 9:**
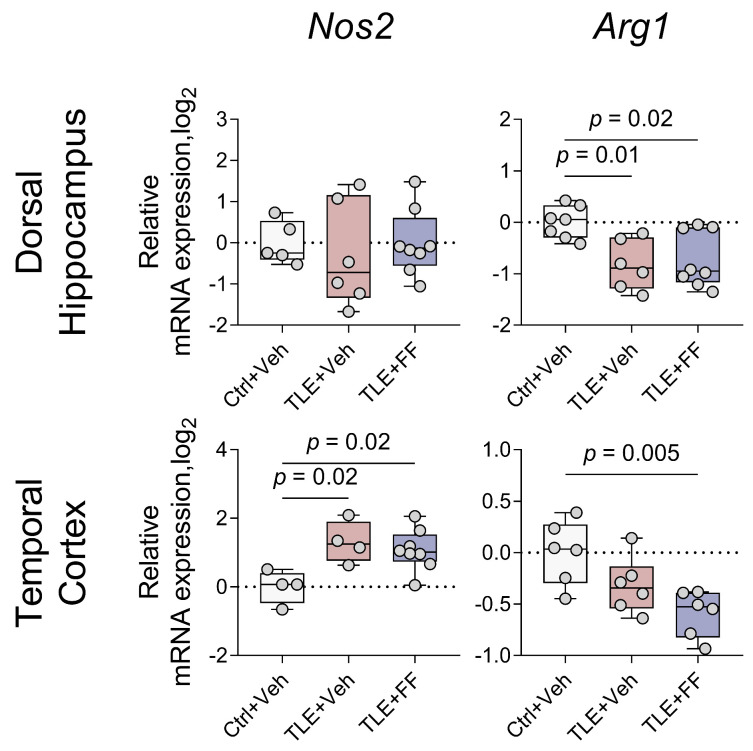
Changes in relative gene expression of microglial polarization markers *Nos2* (M1 phenotype) and *Arg1* (M2 phenotype) in the dorsal hippocampus and temporal cortex of rats. The analysis was performed on day 8 after seizure induction. Ctrl+Veh (*n* = 7), control rats without treatment; TLE+Veh (*n* = 6), TLE rats; TLE+FF (*n* = 8), Fenofibrate-treated TLE rats. One-way ANOVA, only statistically significant results are shown (*p* < 0.05), DH: *Arg1*—F_(2,18)_ = 6.5, *p* = 0.007; TC: *Nos2*—F_(2,13)_ = 6.2, *p* = 0.01; *Arg1*—F_(2,15)_ = 7.3, *p* = 0.006. Full results of one-way ANOVA are shown in Table A3. Significant differences between groups according to Sidak post hoc test are indicated in the figure. Data are presented as individual values (circles) with the minimum, the maximum, the sample median, and the first and third quartiles.

We analyzed the expression of genes conventionally used as markers for A1 (*Lcn2*, *Gbp2*) and A2 (*Ptx3*, *S100a10*) astrocyte phenotypes [48,50], as well as M1 (*Nos2*) and M2 (*Arg1*) microglial phenotypes [51,52,53] in the DH and TC (Figure 8 and Figure 9).

Pilocarpine-induced status epilepticus resulted in a significant upregulation of mRNA levels for both A1 and A2 astrocyte phenotype markers in both brain regions (Figure 8). One-way ANOVA revealed a significant differences on the gene expression of *Lcn2*, *Ptx3*, and *S100a10*. In the DH, post hoc analysis confirmed that the TLE+Veh group exhibited significantly higher expression of *Lcn2* and *S100a10* compared to the Ctrl+Veh group. In the TC, mRNA levels of *Lcn2*, *Ptx3*, and *S100a10* were significantly elevated in the TLE+Veh group compared to controls.

Gene expression of these genes remained elevated following fenofibrate administration (Figure 8). In the DH, the TLE+FF group showed significantly higher expression of *Lcn2* and *S100a10* compared to the Ctrl+Veh group. Notably, fenofibrate treatment induced a significant increase in the expression of the A2 marker *Ptx3* in the DH (TLE+FF vs. Ctrl+Veh, *p* < 0.05). In the TC, the TLE+FF group exhibited significantly higher mRNA levels of *Lcn2*, *Ptx3*, and *S100a10* compared to the Ctrl+Veh group. No significant differences in the expression of *Gbp2* were detected between groups in either brain region.

Analysis of microglial polarization markers revealed region-specific alterations (Figure 9). One-way ANOVA showed a significant effect of treatment on the expression of *Arg1* in the DH and on the expression of *Nos2* and *Arg1* in the TC. Post hoc analysis indicated that in the DH, the expression of *Arg1* was significantly lower in both the TLE+Veh and TLE+FF groups compared to the Ctrl+Veh group. In the TC, the TLE+Veh group showed significantly higher expression of *Nos2* compared to controls. Furthermore, fenofibrate administration significantly reduced the expression of *Arg1* in the TC (TLE+FF vs. Ctrl+Veh, *p* < 0.01).

These results demonstrate that fenofibrate administration during the latent phase enhances the expression of the neuroprotective A2 astrocyte marker *Ptx3* in the DH but reduces the expression of the M2 microglia marker *Arg1* in the TC, indicating a complex, region-specific modulation of glial phenotype polarization.

### 2.8. Fenofibrate Attenuates the Inflammatory Response in the Temporal Cortex but Not in the Dorsal Hippocampus

At the next stage of the work, the expression of genes of pro-inflammatory factors (the main component of the inflammasome *Nlrp3*, interleukin-1β *Il1b*) and the anti-inflammatory cytokine (interleukin-1β receptor antagonist *Il1rn*) was studied in the dorsal hippocampus and temporal cortex of rats during the latent phase of the lithium-pilocarpine model. Both clinical and preclinical studies consistently reveal activated inflammatory signaling in microglia and astrocytes during epilepsy, where these cells critically regulate neuroinflammatory cascades through their dynamic release of immunomodulatory cytokines [54,55,56,57]. The interleukin-1β signaling pathway serves as a key regulator of neuroinflammation, triggering robust inflammatory responses. Our investigation focused on gene expression patterns of key molecular components in this pathway, which demonstrates early activation within seizure-generating brain regions in post-status epilepticus models [58].

**Figure 10:**
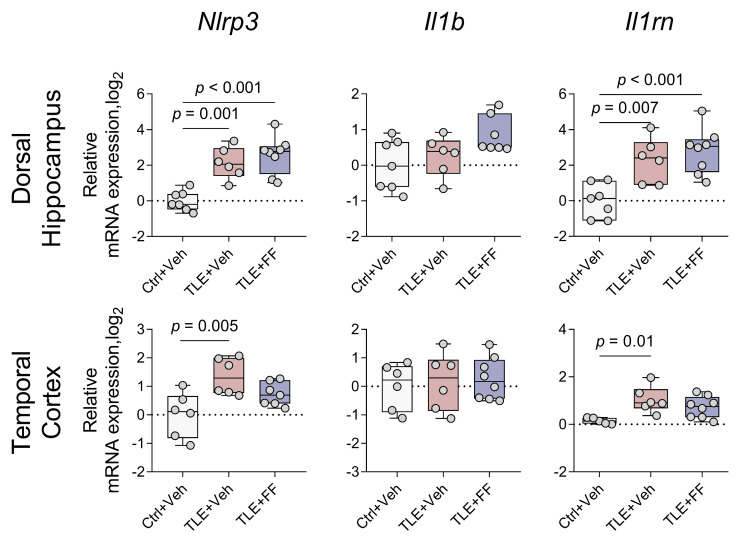
Changes in relative gene expression of *Nlrp3*, *Il1b* and *Il1rn* in the dorsal hippocampus and temporal cortex of rats. The analysis was performed on day 8 after seizure induction. Ctrl+Veh (*n* = 7), control rats without treatment; TLE+Veh (*n* = 6), TLE rats; TLE+FF (*n* = 8), Fenofibrate-treated TLE rats. One-way ANOVA, only statistically significant results are shown (*p* < 0.05), DH: *Nlrp3*—F_(2,18)_ = 17.826, *p* < 0.001; *Il1rn*—F_(2,18)_ = 11.648, *p* = 0.001; TC: *Nlrp3*—F_(2,16)_ = 5.82, *p* = 0.01; *Il1rn*—F_(2,16)_ = 7.153, *p* = 0.006. Full results of one-way ANOVA are shown in Table A3. Significant differences between groups according to Sidak post hoc test are indicated in the figure. Data are presented as individual values (circles) with the minimum, the maximum, the sample median, and the first and third quartiles.

Pilocarpine-induced status epilepticus resulted in a significant upregulation of the inflammasome gene *Nlrp3* in both brain regions (Figure 10). Post hoc analysis confirmed that *Nlrp3* mRNA levels were significantly higher in the TLE+Veh group compared to the Ctrl+Veh group in both the DH and TC.

Similarly, the expression of the interleukin-1 receptor antagonist gene *Il1rn* was significantly increased in both regions. Post hoc tests revealed that *Il1rn* expression was significantly elevated in the TLE+Veh group compared to the Ctrl+Veh group in the DH and TC.

Fenofibrate administration did not attenuate the seizure-induced upregulation of *Nlrp3* or *Il1rn* in the DH. Post hoc analysis indicated that the TLE+FF group exhibited significantly higher mRNA levels of *Nlrp3* and *Il1rn* compared to the Ctrl+Veh group (*p* < 0.001). On the contrary, fenofibrate treatment prevented the pilocarpine-induced upregulation of *Nlrp3* and *Il1rn* in the TC. The mRNA levels of both genes in the TLE+FF group were comparable to those in the Ctrl+Veh group and were not significantly different from the TLE+Veh group.

Statistical analysis revealed no significant changes in *Il1b* gene expression in any of the experimental groups. Although one-way ANOVA indicated a borderline significant main effect in the DH (F_(2,17)_ = 3.675, *p* = 0.047), post hoc testing did not confirm any significant pairwise differences between the control and experimental groups. In the TC, the changes were also statistically non-significant.

These results demonstrate a region-specific modulation of neuroinflammation by fenofibrate. In the TC, fenofibrate treatment effectively normalized the pilocarpine-induced increase in the expression of *Nlrp3* and *Il1rn*, as levels in the TLE+FF group were not significantly different from the control group. Conversely, fenofibrate failed to attenuate the increased expression of these genes in the DH.

### 2.9. Fenofibrate Did Not Affect the Expression of Trophic Factor Genes

**Figure 11:**
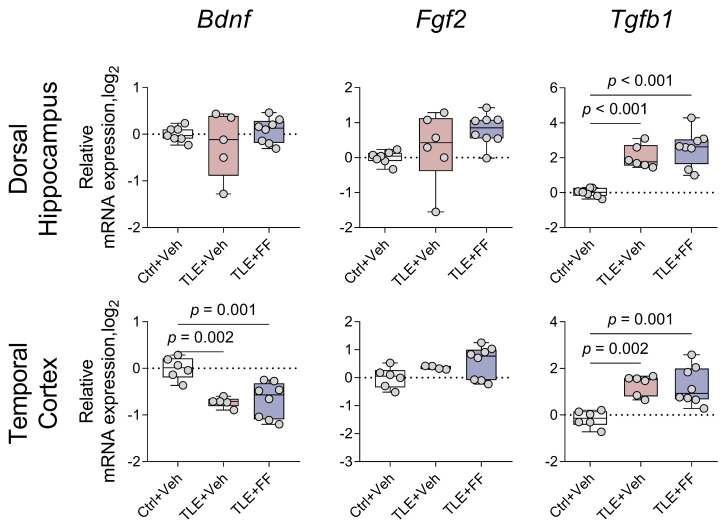
Changes in relative gene expression of trophic factors *Bdnf*, *Fgf2* and *Tgfb1* in the dorsal hippocampus (DH) and temporal cortex (TC) of rats. The analysis was performed on day 8 after seizure induction. Ctrl+Veh (*n* = 7), control rats without treatment; TLE+Veh (*n* = 6), TLE rats; TLE+FF (*n* = 8), Fenofibrate-treated TLE rats. One-way ANOVA or Welch ANOVA, only statistically significant results are shown (*p* < 0.05), DH: *Tgfb1*—F_(2,18)_ = 23.98, *p* < 0.001; TC: *Bdnf*—F_(2,9.8)_ = 21.84, *p* < 0.001; *Tgfb1*—F_(2,11.2)_ = 22.63, *p* < 0.001. Full results of one-way ANOVA or Welch ANOVA are shown in Table A3. Significant differences between groups according to Sidak or Games-Howell multiple comparisons tests are indicated in the figure. Data are presented as individual values (circles) with the minimum, the maximum, the sample median, and the first and third quartiles.

Trophic factors are known to be involved in the regulation of synaptic plasticity and neuroinflammation [59,60]. Epileptogenesis involves dynamic changes in neurotrophic factor expression, including increased production of BDNF, FGF2, and TGFβ1, which contribute to both pathological and compensatory neural remodeling [61]. We analyzed the expression of trophic factor genes (*Bdnf*, *Fgf2*, *Tgfb1*) in this model (Figure 11).

Pilocarpine-induced epileptogenesis significantly altered the expression of specific trophic factors in a region-specific manner (Figure 11). In the TC, one-way ANOVA revealed a substantial decrease in *Bdnf* mRNA levels, with post hoc analysis confirming significantly lower expression in both the TLE+Veh and TLE+FF groups compared to controls. Conversely, *Tgfb1* expression was significantly increased in the TC and DH, with both epileptic groups showing elevated levels compared to controls. No significant alterations were observed in *Fgf2* expression in either brain region, and *Bdnf* levels remained unchanged in the DH across all experimental groups.

Critically, fenofibrate administration failed to affect any of these seizure-induced alterations in trophic factor gene expression. In both brain regions and for all genes examined, the TLE+FF group did not differ significantly from the TLE+Veh group, indicating that fenofibrate treatment did not modulate the expression of these neurotrophic factors during the latent phase of epileptogenesis.

## 3. Discussion

Our study provides the first comprehensive evidence that the PPARα agonist fenofibrate exerts a complex, region-specific modulation of neuroinflammation and synaptic plasticity during the latent phase of the lithium-pilocarpine model of temporal lobe epilepsy. While conferring significant behavioral benefits, cortical anti-inflammatory effects, and a positive impact on the expression of cortical ionotropic glutamate receptor subunits, its action in the hippocampus remains ambiguous, highlighting both its therapeutic potential and its limitations.

### 3.1. Fenofibrate Decreased Blood Levels of Short-Chain Fatty Acids

It is well established that the hallmark features of epilepsy are excitation/inhibition imbalance [62] and neuroinflammation [63]. Various types of PPARs, particularly PPARα, regulate cellular energy metabolism, inflammation, and neuroprotection [7]. Through PPARα-mediated regulation of inflammatory pathways and cellular metabolism, fenofibrate, PPARα agonist, exhibits combined neuroprotective and antidiabetic properties capable of ameliorating both Alzheimer’s disease pathology and type 2 diabetes complications [64]. Furthermore, PPARα in the central nervous system regulates neuronal activity by modulating nicotinic acetylcholine receptors [20], suggesting that PPARα agonists may alter excitation/inhibition balance through this mechanism. In this regard, we hypothesized that fenofibrate might exert a beneficial effect on the course of epileptogenesis.

In our study, we primarily assessed blood concentrations of short-chain fatty acids (SCFAs), which are endogenous ligands of PPARs [8], in rats following fenofibrate administration. Existing research provides minimal evidence regarding influence of fenofibrate on systemic SCFA profiles in animal models. Previous research demonstrated that 7-day fenofibrate administration during a fructose-enriched diet reduced blood triglyceride and free fatty acid levels [65]. However, this study lacked a normal diet control group and did not assess SCFA concentrations. In a mouse model of high-fat diet, chronic fenofibrate treatment showed no effect on propionic or butyric acid levels in feces and blood serum [22], though this work did not evaluate larger SCFA molecules. In our study, fenofibrate administration significantly decreased blood concentrations of 2-methylpropanoic, butyric, pentanoic, and hexanoic acids in rats. Notably, according to the two-way ANOVA performed the interaction between TLE and Treatment factors was not significant for any SCFA, indicating that the effect of fenofibrate was consistent and independent of the disease state. These findings suggest activation of fatty acid metabolism genes, consistent with PPARα agonism by fenofibrate, but do not support the hypothesis that elevated systemic SCFA levels mediate its protective effects. In addition, the observed reduction in SCFA levels could be mediated by decreased intestinal production due to activation of intestinal PPARα by fenofibrate and subsequent intensification of host antimicrobial activity of the local immune or epithelial cells. Although, this assumption needs further verification, it could be an example of negative feedback mechanism of host control of intestinal microbial content as SCFA apparently are the important ligands of PPARα in the large intestine [66].

It is important to note that, according to the two-way ANOVA performed, the TLE factor also significantly affects the levels of butyric and 2-methylpropanoic acid. It can be assumed that pilocarpine-induced seizures may lead to disturbances in the content of SCFAs, which could be one of the mechanisms behind impaired energy balance in epilepsy [67]. This link between seizure-induced SCFA alterations and energy metabolism provides a potential mechanistic foundation for the efficacy of the ketogenic diet, which is thought to compensate for such metabolic disturbances [68].

Beyond serving as PPARα ligands, the reduction in SCFAs observed in our study, particularly butyrate, may have broader implications. Butyrate is a well-known inhibitor of histone deacetylases (HDACs) [69]. HDAC inhibition has been shown to possess anticonvulsant and neuroprotective effects in various disease models, including epilepsy [70,71,72]. Therefore, the fenofibrate-induced decrease in circulating butyrate could potentially influence epigenetic mechanisms. This interplay between PPARα activation and HDAC inhibition via SCFA modulation warrants further investigation in the context of epileptogenesis.

### 3.2. Fenofibrate Exerts Region-Specific Effects on Gene Expression of Glutamate Receptors and Transporters

It is established that epileptogenesis involves altered expression of numerous genes participating in various signaling pathways [73]. In our study, we firstly investigated changes in the expression of genes encoding subunits of ionotropic glutamate receptors. Pilocarpine-induced seizures lead to reduced gene expression of NMDA and AMPA receptor subunits in the dorsal hippocampus and temporal cortex of rats. It is known that while pilocarpine-induced M1 receptor activation initiates status epilepticus by disrupting excitation/inhibition balance [25], seizure persistence becomes independent of cholinergic signaling [74]. Studies have confirmed the involvement of the glutamatergic system, particularly NMDA receptors, in sustaining seizure activity [25,75,76]. This is consistent with observed increases in hippocampal glutamate levels following seizure onset [76]. Consequently, the observed downregulation of NMDA and AMPA receptor subunit genes likely represents a compensatory mechanism to attenuate neuronal hyperexcitability [77]. These changes likely form the basis for the impairment in LTP induction we demonstrated earlier [78]. Fenofibrate treatment restored the downregulated NMDA (*Grin1*, *Grin2a*) and AMPA (*Gria1*, *Gria2*) receptor subunit gene expression to control levels only in the temporal cortex. Conversely, in the dorsal hippocampus, fenofibrate administration resulted in further suppression of *Grin2a*, *Grin2b*, *Gria1*, *Gria2* gene expression. The fenofibrate-induced modulation of glutamate receptor subunit gene expression we observed has no direct precedents in published literature. Fenofibrate’s region-specific modulation likely represents an adaptive neuroprotection: it restores receptor levels in the temporal cortex to normalize function but further suppresses them in the vulnerable hippocampus to limit excitotoxic damage.

No corresponding changes in NMDA and AMPA receptor subunit protein levels were detected. This may be due to the fact that a temporal decoupling of transcription and translation is frequently observed for numerous proteins under pathological conditions [79,80]. In particular, such transcript-protein discordance was demonstrated for NMDA receptor subunit genes in the neuroinflammatory model [81] and for NMDA and AMPA subunit receptor genes in the model of pentylenetetrazole-induced acute seizures [82].

In our study, we also analyzed the expression of glutamate transporters and glutamine synthetase genes during the latent phase of the model. In epilepsy, the production of mRNA of major glial transporters typically decreases [83]. However, we identified increased expression of genes encoding EAAT1 and EAAT2, as well as glutamine synthetase, which likely reflects a compensatory mechanism aimed at reducing hyperexcitation. These changes were observed exclusively in the dorsal hippocampus. We had previously demonstrated reduced EAAT2 protein levels in the dorsal hippocampus during the latent phase [42], which supports the hypothesis that the increased expression of the EAAT2-encoding gene we observed represents a compensatory response. Fenofibrate did not affect the observed changes in expression of astrocytic glutamate transporter and glutamine synthetase genes. However, fenofibrate treatment reduced expression of the neuronal glutamate transporter gene *Slc1a1*. Data regarding changes in the expression of this transporter in the literature are contradictory. On the one hand, it is suggested that a reduction in the production of this transporter is a feature of epilepsy [84]. On the other hand, studies have shown a localized increase in EAAT3 expression in epilepsy [85]. Such a local increase in EAAT3 production was demonstrated in dentate gyrus granule cells, also in the pilocarpine rat model. In our study, we did not separate hippocampal cell types; it is likely that the expression of this transporter is heterogeneous across the hippocampus, which complicates the assessment of fenofibrate’s effect in this case.

### 3.3. Effect of Fenofibrate on Gene Expression of Glial and Neuroinflammation Factors

Astro- and microgliosis, along with elevated expression of *Gfap* and *Aif1* genes, are well-documented feature of both clinical temporal lobe epilepsy and the lithium-pilocarpine model [42,45,86,87]. Beyond being a consequence of epileptogenesis, glial activation actively drives disease progression [88]. In our study, we detected increased expression of these genes in both the hippocampus and temporal cortex of rats in the latent phase of the model. GFAP protein level was similarly elevated in the temporal cortex. Expression of the *S100b* gene, another astrocytic marker, was increased only in the dorsal area of the hippocampus. Although fenofibrate did not have a significant effect on the expression of these genes, the expression of *S100b* genes in the dorsal hippocampus and *Aif1* in the temporal cortex was slightly reduced, as no significant differences were found between the control group and the TLE+FF group. Notably, fenofibrate has been shown to reduce elevated GFAP levels in the hippocampus of experimental animals in non-epileptic models [89,90]. However, in addition to the use of other experimental models, the discrepancies may be related to different treatment protocols: Erdogan et al. (2024) [89] and Villavicencio-Tejo et al. (2021) [90] employed longer-term fenofibrate treatment and administered it orally.

In this study, we analyzed the expression of genes marking various astrocyte and microglia phenotypes. Increased expression was detected for markers of both A1 astrocyte phenotype and A2 phenotype in the dorsal hippocampus (*Lcn2*, *S100a10*) and temporal cortex (*Lcn2*, *Ptx3*, *S100a10*). Fenofibrate administration also led to increased *Ptx3* mRNA production in the dorsal hippocampus, which may represent a beneficial effect of the drug. This gene is considered a marker of the A2 neuroprotective astrocyte phenotype [48]; therefore, this change may promote the predominance of anti-inflammatory processes. Although PPARα widely represented on astroglia, data regarding fenofibrate-induced modifications of their polarization status are absent or extremely limited [91]. Existing studies primarily focus on reducing inflammatory activation, without providing conclusive evidence about modifications of polarization processes within the astroglial response.

Changes in the expression of microglial phenotype marker genes were multidirectional. In the dorsal hippocampus, only a decrease in the *Arg1* gene, which is a marker of the M2 microglial state [47], was observed. This change may promote epileptogenesis by reducing microglial anti-inflammatory responses. Fenofibrate did not affect this alteration. In the temporal cortex, increased expression of the *Nos2* gene, a marker of the M1 microglial state [47], was noted, which may also contribute to epileptogenesis. Fenofibrate treatment did not influence *Nos2* gene expression. Data obtained from the amyotrophic lateral sclerosis model demonstrated reduced iNOS production [92], although in that study the fenofibrate dose used was 4 times higher than in our research. Moreover, in our study fenofibrate led to decreased *Arg1* mRNA production, likely representing a negative effect of the drug. However, in experimental models of multiple sclerosis, fenofibrate reduced expression of IL-2, IL-6 and key Toll-like receptor signaling components (including CD14), thereby decreasing microglial activation and pro-inflammatory mediator secretion [93]. Thus, it remains possible that fenofibrate modulated other pro-inflammatory mediators not assessed in our study, potentially compensating for the *Arg1* downregulation and preventing adverse outcomes.

We examined the expression of key interleukin-1β signaling pathway genes in rat brain during the latent phase of the model. Increased mRNA production of *Nlrp3* and *Il1rn* was detected, consistent with previously reported findings [27,44,94]. The expression of the interleukin-1β encoding gene remained unchanged. Although fenofibrate was anticipated to exert more substantial effects on neuroinflammatory marker expression due to PPARα’s known anti-inflammatory properties [13], its administration only modestly reduced *Nlrp3* and *Il1rn* gene expression in the temporal cortex, with no observable effects in the dorsal hippocampus.

Trophic factors are important regulators of both neuroinflammation and synaptic plasticity [59,60]. We studied the gene expression levels of several trophic factors (*Bdnf*, *Fgf2*, *Tgfb1*) and identified increased *Tgfb1* expression in both examined rat brain structures and decreased *Bdnf* expression in the temporal cortex. We had previously demonstrated increased *Tgfb1* gene expression during the latent phase of the model [44], which likely represents a characteristic feature of this stage in the lithium-pilocarpine paradigm. Given that elevated BDNF expression during the early post-seizure period is considered epileptogenic [95,96,97], the decreased *Bdnf* expression we observed likely represents a compensatory mechanism. Fenofibrate did not affect the expression of trophic factors.

### 3.4. Regional-Specific Effects of Fenofibrate on the Expression of the Investigated Genes

In the course of this study, we found that fenofibrate exerted region-specific effects on the expression of the investigated genes. Specifically, fenofibrate restored the mRNA levels of the majority of the investigated ionotropic glutamate receptor subunits, as well as neuroinflammatory factors, in the temporal cortex. In the hippocampus, however, fenofibrate had no effect on neuroinflammatory factors and further reduced the expression of NMDA and AMPA receptor subunit genes. At the same time, it is important to note that a beneficial effect was observed regarding glial phenotype markers in the dorsal hippocampus: fenofibrate led to an increase in the production of mRNA for the anti-inflammatory A2 astrocyte phenotype marker (*Ptx3*). It is well-established that the hippocampus is more vulnerable to the consequences of seizures than the cortex, particularly to neuroinflammation [25,98]. In our study, we observed that fenofibrate reduced the expression of neuroinflammatory factors only in the cortex, while inflammatory processes persisted in the hippocampus. It is known that neuroinflammation can contribute to seizure development by enhancing excitatory glutamatergic transmission and reducing inhibitory GABAergic transmission [99]. Thus, the fenofibrate-induced downregulation of ionotropic receptor subunit genes, along with the increased *Ptx3* mRNA production in the dorsal hippocampus, may also be compensatory. However, this pronounced downregulation of ionotropic glutamate receptor genes may potentially lead to future cognitive deficits. A more effective strategy for the dorsal hippocampus might be to target a greater reduction in neuroinflammation.

### 3.5. Attenuation of Epilepsy-Associated Behavioral Comorbidities by Fenofibrate

A significant body of research has established a strong association between epilepsy and a higher prevalence of various comorbid conditions [3,100]. In particular, epilepsy is frequently accompanied by significant challenges in cognitive processing, psychiatric health, and social-adaptive behaviors [101]. The lithium-pilocarpine model of epilepsy successfully replicates most comorbid disorders associated with epilepsy [102,103]. In this study we showed that during the latent phase of the lithium-pilocarpine model, animals exhibited increased anxiety-like behavior and reduced exploratory activity. The obtained results correspond to the previously described features of various behavioral impairments in the lithium-pilocarpine model [45,103,104,105,106]. Fenofibrate administration ameliorates anxiety-like behaviors and normalizes exploratory deficits in rats. This positive behavioral outcome is consistent with our observed reduction in neuroinflammation and the restoration of NMDA and AMPA receptor subunit mRNA levels in the temporal cortex.

### 3.6. Limitations of the Study

Several other limitations of the study should be acknowledged. To maintain data consistency, we exclusively used male animals, given the established sex-dependent variability in this model [107]. We tested the effects of fenofibrate during the latent phase; consequently, its effect on spontaneous recurrent seizures was not studied. Furthermore, a single fenofibrate dose was used throughout the study. Although this dose showed efficacy in modulating SCFA levels, its impact on epileptogenesis was mild. Given the regional differences we found in the effects of fenofibrate, it is likely that these effects will change with dose. To better characterize fenofibrate effects on neuroinflammation and astrocyte/microglia states, future studies should assess a broader panel of molecular markers. The effect of fenofibrate on antioxidant stress and mitochondrial dysfunction, which are also known consequences of seizures [29], was not assessed in this study.

It is also important to note that in our study, the Ctrl+FF group was included only in the analysis of SCFA levels. This precludes a definitive assessment of fenofibrate’s effects in the healthy brain. However, published evidence suggests that fenofibrate administration in control rodents does not significantly alter the expression of genes or proteins related to neuroinflammation or glial reactivity that are not directly involved in lipid metabolism [90,108]. For instance, Villavicencio-Tejo et al. reported no effect of fenofibrate on GFAP protein levels in the hippocampus of control rats [90]. Furthermore, Yaribeygi et al. showed that fenofibrate’s effects on inflammatory markers were specific to a disease model (diabetic nephropathy) and were not observed in control animals [108]. This supports our study’s focus on the therapeutic potential of fenofibrate in the disease context (TLE group). Nonetheless, we acknowledge that unforeseen, region-specific effects in healthy tissue cannot be entirely ruled out. Furthermore, given the role of SCFAs and HDACs in regulating mitochondrial function and protein acylation, the observed changes might exert complex secondary effects on cellular metabolism and signaling, which warrant further investigation. In particular, the consequences of the reduction in butyric acid and 2-methylpropanoic acid require further study, as the levels of these acids were influenced by both the TLE factor and the Treatment factor according to the two-way ANOVA.

While this study was conducted in a preclinical model, our findings suggest that fenofibrate, an already FDA-approved drug with a known safety profile, could be repurposed as a complementary therapy for TLE. Its ability to reduce anxiety and improve exploratory behavior addresses critical comorbid conditions that significantly impact patients’ quality of life. However, our data also caution that a simple translation to humans is not straightforward. The observed region-specific effects, particularly the potential exacerbation of hippocampal glutamate receptor downregulation, highlight the necessity for further studies to optimize dosing regimens and timing of administration.

## 4. Materials and Methods

### 4.1. Experimental Design

The study employed the lithium-pilocarpine model of temporal lobe epilepsy (TLE), in which, just as in human pathology, epileptogenesis involves several stages: status epilepticus, a latent period without seizure manifestations, and a chronic phase characterized by the occurrence of spontaneous recurrent seizures [25].

The Wistar rats used in this study were sourced from the in-house breeding colony of the Sechenov Institute of Evolutionary Physiology and Biochemistry, Russian Academy of Sciences (St. Petersburg, Russia). Eight-week-old male Wistar rats were administered: (1) 127 mg/kg LiCl (Sigma-Aldrich, St. Louis, MO, USA); (2) after 23 h, 1 mg/kg scopolamine methyl bromide (Sigma-Aldrich) to prevent peripheral effects of pilocarpine; (3) after another 1 h-the muscarinic receptor agonist pilocarpine (Sigma-Aldrich). Pilocarpine was administered incrementally at 10 mg/kg every 30 min until development of stage 4 seizures according to the modified Racine scale [109]. The total pilocarpine dose was 20–40 mg/kg. Rats that did not develop seizures after the fourth injection (40 mg/kg) were excluded from the experiment. Seizures were stopped after 90 min by administration of 200 mg/kg chloral hydrate (Sigma-Aldrich). Control animals received saline. We have previously shown that this protocol induces TLE in most experimental animals [45]. An 1 h and a half after status epilepticus, half of the rats received 100 mg/kg fenofibrate (FF, Sigma-Aldrich) in a volume of 0.2 mL/100 g weight, the remaining rats received an equivalent volume of DMSO, which was used as a solvent for FF. All described injections were administered to rats intraperitoneally.

**Figure 12:**
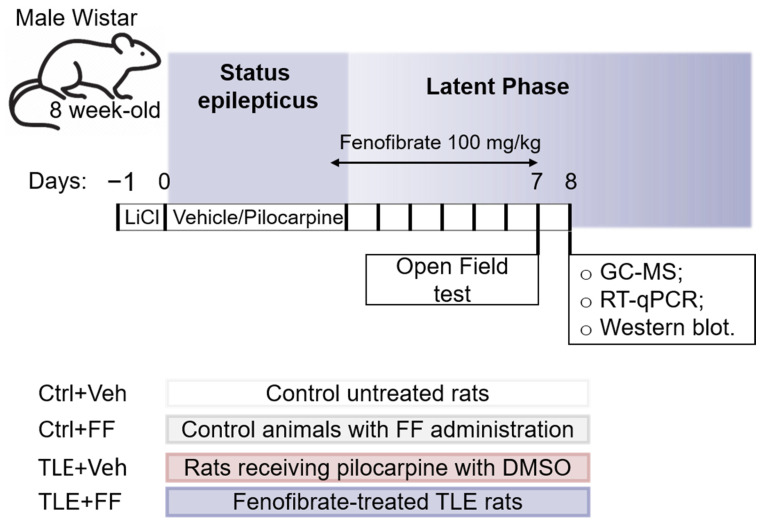
Schematic representation of the experimental design. GC-MS, gas chromatography with mass spectrometric detection; RT-qPCR, reverse transcription followed by quantitative polymerase chain reaction.

Thus, four experimental groups were randomly formed: (1) Ctrl+Veh—control untreated rats; (2) Ctrl+FF—control animals with FF administration; (3) TLE+Veh—rats receiving pilocarpine with DMSO; (4) TLE+FF—fenofibrate-treated TLE rats (Figure 12). To ensure robust findings while adhering to the 3R principles, sample sizes were determined a priori using G*Power 3.1 (α = 0.05, power = 0.8) based on effect sizes from prior work. To control for litter effects, pups from each litter were distributed across all experimental groups.

The drugs were administered for 7 days (the first injection–24 h after pilocarpine administration). On the last day of fenofibrate administration (23 h after the previous injection), the rats’ behavior was assessed in the Open Field test (behavioral hyperexcitability, anxiety level and exploratory activity), then after 24 h brain and blood samples were collected for biochemical studies.

The animals were kept under standard conditions with free access to water and food. During the first week after seizure induction, due to the animals’ severe condition, they were fed wet food (fruits, porridge). Body weight was measured daily. Animals that lost significant weight received intramuscular injections of 15% glucose solution. The experiments were conducted in accordance with the Guidelines of the Committee for the Care and Use of Animals of the I.M. Sechenov Institute of Evolutionary Physiology and Biochemistry, the EU Directive 2010/63/EU on animal experimentation, and the ARRIVE guidelines. All outcome assessments were performed by investigators blinded to the group allocation. This includes behavioral testing (The Open Field test) and biochemical assays. Coded samples were used to ensure blinding.

### 4.2. Behavioral Testing

Behavioral testing was performed on day 7 following the administration of pilocarpine (the latent phase of the model). Test was conducted in the evening during the period of natural peak activity in rats (from 17:00 to 23:00).

The Open Field test was used to assess exploratory and locomotor activity, as well as anxiety levels. The experimental arena for conducting the test was a round open field enclosed by a perimeter wall. The field diameter is 1 m, and the wall height is 30 cm. The floor of the setup contained 16 round holes with a diameter of 4 cm and a depth of 3 cm each. The illumination of the experimental zone was 8 lux. The experimenter placed the rat in the center, after which the animal was allowed to explore the open field for 5 min. After each animal, the setup was cleaned with a 15% ethanol solution. The animal’s behavior was recorded by two cameras: one was positioned above the setup, the other filmed from a side view. The video recording from the top camera was subsequently used to analyze the distance traveled and time spent in the central zone (anxiety indicator). The video recording from the side camera provided a close-up image; it was used to determine the frequency and duration of individual behavioral patterns, locomotor activity indicators (locomotion time), exploratory activity (time spent investigating holes and rearing with support), and anxiety levels (autogrooming time).

### 4.3. Biochemical Methods

On day 8 after status epilepticus (latent phase of the model), the rats were anesthetized with an Isoflurane (Laboratorios Karizoo, Barcelona, Spain) and decapitated for the collection of blood and brain samples. Blood was collected to assess the level of short-chain fatty acids in plasma using Gas Chromatography-Mass Spectrometry (GC-MS). The brain was extracted and frozen at a temperature of −80 °C. Using an OTF5000 cryostat microtome (Bright Instrument, Luton, UK), the dorsal hippocampus and temporal cortex were isolated according to the rat brain atlas until the subsequent biochemical studies could be conducted. The expression of genes involved in epileptogenesis was evaluated at the mRNA and protein levels by reverse transcription followed by polymerase chain reaction (RT-qPCR) and Western blotting, respectively.

#### 4.3.1. GS-MS

Blood was collected into K_2_EDTA-containing Lab-Vac vacuum tubes (Shandong Chengwu Medical Products Factory, Heze, China). Samples were centrifuged at 3000× *g* for 15 min at +4 °C. Plasma was collected, aliquoted (200 µL) into Eppendorf tubes, and stored at −20 °C until analysis. Sample preparation for GC-MS analysis was performed by adding 30 μL of 5 M HCl, 200 μL of blood plasma, and 300 μL of diethyl ether (DEE) to a vial. The mixture was vortexed for 1 min, centrifuged at 5000 rpm for 5 min, and the ether layer was transferred to a vial containing anhydrous sodium sulfate. The extraction procedure (addition of 300 μL DEE, vortexing, centrifugation, transfer of ether layer) was repeated twice more, yielding a total of three extractions. A 200 μL aliquot of the combined ether extracts was transferred to a clean vial. Then, 10 μL of N,O-Bis(trimethylsilyl)trifluoroacetamide (BSTFA) was added, and the mixture was incubated at +70 °C for 30 min to form trimethylsilyl (TMS) derivatives of the target SCFAs: propanoic acid, 2-methylpropanoic acid, butyric acid, 3-methylbutanoic acid, pentanoic acid, 4-methylvaleric acid, hexanoic acid, and heptanoic acid.

Derivatized samples were analyzed using a Chromatec Crystal 5000 GC-MS system with a mass spectrometric detector and a DASH 3D Universal autosampler (Chromatec, Yoshkar-Ola, Russia) equipped with an HP-5MS capillary column (30 m × 0.250 mm × 0.25 μm; Agilent Technologies, Santa Clara, CA, USA). Chromatographic conditions: Helium carrier gas at 1 mL/min; Transfer line: +300 °C; Ion source: +250 °C; Injector: +250 °C (split mode, 1:10 ratio, injection volume: 1 μL). Oven temperature program: +40 °C (hold 2 min), ramp to +150 °C at +15 °C/min (hold 1 min), ramp to +300 °C at +30 °C/min (hold 5 min). Mass spectrometric detection was performed in targeted Selected Ion Monitoring mode, monitoring characteristic ions for the TMS derivatives of each target SCFA: *m*/*z* 131 (propanoic acid-TMS), *m*/*z* 145 (2-methylpropanoic acid-TMS, butyric acid-TMS), *m*/*z* 159 (3-methylbutanoic acid-TMS, pentanoic acid-TMS), *m*/*z* 173 (4-methylvaleric acid-TMS, hexanoic acid-TMS), and *m*/*z* 187 (heptanoic acid-TMS). Data acquisition and processing were performed using Chromatec Analyst software version 3.1 (Chromatec). Quantification of individual SCFAs was achieved using external calibration curves. Calibration standards containing known concentrations of each target acid were subjected to the same sample preparation procedure (derivatization with BSTFA), and analyzed identically to the plasma samples. Analyte concentrations in plasma samples were determined by interpolating peak areas against the respective calibration curves.

#### 4.3.2. RT-qPCR

Total RNA was extracted using the ExtractRNA reagent (Evrogen, Moscow, Russia) according to the manufacturer’s instruction. To eliminate possible genomic DNA contamination, RQ1 DNase (Promega, Madison, WI, USA) was applied to the samples according to the manufacturer’s instructions. RNA concentration and purity were assessed spectrophotometrically using NanoDrop Lite Spectrophotometer (Thermo Fisher Scientific, Waltham, MA, USA). This involved measuring the absorbance at 260 nm and calculating the 260/280 nm absorbance ratio. Reverse transcription was conducted in a 25 μL volume using M-MLV reverse transcriptase (100 units per 1 μg RNA; Evrogen) according to the manufacturer’s protocol. For the reaction, 2 μg of total RNA, 2 μg of oligo-dT primers, and 1 μg of random 9-mer primers were taken.

The qPCR procedure was performed in a 10 µL volume consisting 0.8 µL cDNA, 0.75 units of TaqM-polymerase (Alkor Bio, St. Petersburg, Russia), 3.5 mM Mg^2+^, specific forward and reverse primers, and hydrolysis (TaqMan) probes in assay-dependent concentrations (Table A1). All nucleotides were synthesized by DNA Synthesis Ltd. (Moscow, Russia).

Tetraplicated PCRs were conducted in a C1000 Touch thermal cycler combined with a CFX384 Touch™ Real-Time PCR Detection System (Bio-Rad, Hercules, CA, USA), simultaneously with no template control samples. The following multiplexes were used in this work: *Actb* + *Gapdh* + *B2m*, *Rpl13a* + *Ppia* + *Sdha*, *Hprt1* + *Pgk1* + *Ywhaz*, *Grin1* + *Grin2a*, *Grin2b* + *Gria1* + *Gria2*, *Gfap* + *Slc1a2*, *Aif1* + *Nlrp3*, *Tgfb1* + *Slc1a1* + *Slc1a3*, *Lcn2* + *Arg1* + *S100a10*, *Bdnf* + *Fgf2*, *S100b* + *Glul*. PCRs for *Gbp2*, *Il1b*, *Il1rn*, *Ptx3* and *Nos2* were carried out as a singleplexes.

The relative expression of interest genes was calculated using the 2^−ΔΔCt^ method [110]. The data were normalized based on the geometric mean of the three most stable reference genes in the analyzed brain regions: *Pgk1*, *Ppia*, and *Sdha* for the hippocampus; *Ywhaz*, *Actb*, and *Gapdh* for the temporal cortex. The reference genes for each structure were selected in the same way in our previous study [111].

#### 4.3.3. Western Blot

Protein extraction from samples was performed in 200 μL of lysis buffer [112] according to the protocol we described previously [27]. Protein concentration was determined by a modified Lowry method (BSA was used as a standard) [113]. The protein sample was mixed with an equal volume of 2× sample loading buffer (125 mM Tris-HCl pH 6.8, 40% glycerol, 4% SDS, 10% β-mercaptoethanol, 0.02% bromphenol blue) and heated at +70 °C for 15 min.

For electrophoresis, the optimal protein load within the linear range of all antibodies used was pre-determined. To achieve this, tests were carried out with protein dilutions of 2.5 to 15 μg per lane, analyzing both samples from individual groups and composite samples prepared by mixing specimens from each group. Equal volumes of proteins (8 μg) were separated by SDS-PAGE in 7% (for GluN2a, GluN2b, GluA1, GluA2, GFAP) or 10% (for GluN1, EAAT2) polyacrylamide gels under reducing and denaturing conditions with a Thermo Scientific PageRuler Prestained Protein Ladder (10–170 kDa; Thermo Fisher Scientific). Details of the antibody manufacturer and catalog numbers are given in Table A2.

Then, proteins were transferred from the gel onto nitrocellulose membrane (pore diameter 45 μm) by semi-wet transfer with 1× Power Blotter 1-Step Transfer Buffer (Thermo Fisher Scientific) following manufacturer’s instructions. After transfer, the membranes were stained for total lane protein with 0.1% Ponceau S (dissolved in 5% acetic acid) and documented with a ChemiDoc MP imager (Bio-Rad). Blocking was performed in 0.5% skim milk powder solution (Sigma-Aldrich) diluted in PBST. Next step, membranes incubated overnight at +4 °C in primary antibody (specific solution for the assay was prepared in PBST containing 0.05% NaN_3_; see Table A2 for antibody details). The membranes were then washed with PBST four times, incubated in a solution of the secondary antibody (see Table A2) in PBST at room temperature for 10 min and one more time washed in PBST. All washes and immunostaining were performed using the SNAP I.D. 2.0 vacuum blot hybridization system (Merck Millipore, Burlington, MA, USA) according to the manufacturer’s recommendations. Specific proteins were detected using SuperSignal West Pico PLUS chemiluminescent substrate (Thermo Fisher Scientific) and visualized on a ChemiDoc MP imager (Bio-Rad).

Images were analyzed using Image Lab 6.0.1 software (Bio-Rad). Protein expression was normalized to the total protein loading of Ponceau-stained membranes [114] following a total protein normalization method (Bio-Rad). This way of quantitatively analyzing Western blot results is considered preferable [115,116,117,118]. The ratio of the optical densities of the specific protein band to total lane protein was calculated and normalized to the mean of the control group.

### 4.4. Statistical Methods

Statistical analysis was performed using IBM SPSS Statistics 23 (IBM, Armonk, NY, USA) and GraphPad Prism 8.0.1 software (GraphPad Software, San Diego, CA, USA). Outliers were removed using the quartile method. For the analysis of survival curves in the rats was employed the log rank test. The normality of the sample data distribution was assessed with a Shapiro–Wilk test. The equality of variance was checked Leven’s test. A mixed ANOVA was employed to analyze weight dynamics during drug administration. Two-way ANOVA was used to analyze the GC-MS data. For the rest of the data, which had a normal distribution, one-way analysis of variance (ANOVA) or the Welch test was used if the data had uneven variance. Multiple comparisons were performed using the Sidak and Games-Howell post hoc tests. The Kruskal–Wallis test followed by Dunn post hoc test were assessed for non-normally distributed data.

Statistical significance was determined at *p* < 0.05. The values obtained as a result of statistical tests are given in Table A3. The presented PCR data consist as individual values (circles on graphs) with the minimum, the maximum, the sample median, and the first and third quartiles. The rest of the data are presented as averages with a standard deviation, individual values for each rat shown circles.

## Data Availability

The data presented in this study are available upon request from the corresponding author.

## Appendix A

**Table A1:** Primers and probes used in RT-PCR.

Gene Symbol RefSeq Accession Number	Nucleotide Sequences (Forward, Reverse, TaqMan-Probe)	Final Concentration nM	Reference
Housekeeping genes			
*Actb* NM_031144	TGTCACCAACTGGGACGATA GGGGTGTTGAAGGTCTCAAA FAM-CGTGTGGCCCCTGAGGAGCAC-BHQ1	200 200	[119] [120]
*Gapdh* NM_017008	TGCACCACCAACTGCTTAG GGATGCAGGGATGATGTTC R6G-ATCACGCCACAGCTTTCCAGAGGG-BHQ2	200 100	[121]
*B2m* NM_012512	TGCCATTCAGAAAACTCCCC GAGGAAGTTGGGCTTCCCATT ROX-ATTCAAGTGTACTCTCGCCATCCACCG-BHQ1	200 100	[122]
*Rpl13a* NM_173340	GGATCCCTCCACCCTATGACA CTGGTACTTCCACCCGACCTC FAM-CTGCCCTCAAGGTTGTGCGGCT-BHQ1	200 100	[123] [120]
*Sdha* NM_130428	AGACGTTTGACAGGGGAATG TCATCAATCCGCACCTTGTA R6G-ACCTGGTGGAGACGCTGGAGCT-BHQ2	200 100	[124] [120]
*Ppia* NM_017101	AGGATTCATGTGCCAGGGTG CTCAGTCTTGGCAGTGCAGA ROX-CACGCCATAATGGCACTGGTGGCA-BHQ1	200 100	[125]
*Hprt1* NM_012583	TCCTCAGACCGCTTTTCCCGC TCATCATCACTAATCACGACGCTGG FAM-CCGACCGGTTCTGTCATGTCGACCCT-BHQ1	200 100	[126] [120]
*Pgk1* NM_053291	ATGCAAAGACTGGCCAAGCTAC AGCCACAGCCTCAGCATATTTC R6G-TGCTGGCTGGATGGGCTTGGA-BHQ2	200 100	[127] [120]
*Ywhaz* NM_013011	GATGAAGCCATTGCTGAACTTG GTCTCCTTGGGTATCCGATGTC ROX-TGAAGAGTCGTACAAAGACAGCACGC-BHQ1	200 100	[127] [120]
Ionotropic glutamate receptor subunit genes			
*Grin1* NM_017010	GTTCTTCCGCTCAGGCTTTG AGGGAAACGTTCTGCTTCCA FAM-CGGCATGCGCAAGGACAGCC-BHQ1	200 100	[128]
*Grin2a* NM_012573	GCTACACACCCTGCACCAATT CACCTGGTAACCTTCCTCAGTGA FAM-TGGTCAATGTGACTTGGGATGGCAA-BHQ1	200 100	[129]
*Grin2b* NM_012574	CCCAACATGCTCTCTCCCTTAA CAGCTAGTCGGCTCTCTTGGTT FAM-GACGCCAAACCTCTAGGCGGACAG-BHQ1	200 100	[129]
*Gria1* NM_031608	TCAGAACGCCTCAACGCC TGTAGTGGTACCCGATGCCA ROX-TCCTGGGCCAGATCGTGAAGCTAGAAAA-BHQ1	200 100	[130]
*Gria2* NM_017261	CAGTGCATTTCGGGTAGGGA TGCGAAACTGTTGGCTACCT FAM-TCGGAGTTCAGACTGACACCCCA-BHQ1	200 100	[130]
Astrocyte and microglial marker genes			
*Gfap* NM_017009.2	TGGCCACCAGTAACATGCAA CAGTTGGCGGCGATAGTCAT HEX-CGGTCCAAGTTTGCAGACCTCACAG-BHQ2	200 200	[131] [45]
*S100b* NM_013191.2	AAGTCCACACCCAGTCCTCT AGGCTCCTGGTCACCTTTTG HEX-ACACCGAAGCCAGAGAGGACTCCGG-BHQ2	200 100	[44]
*Aif1* NM_017196.3	CAACACACTGCAGCCTCATC AAGCTTTTCCTCCCTGCAAA Cy5-CCCCACCTAAGGCCACCAGCGTCTGA-BHQ3	200 100	[132]
Glutamate-glutamine cycle genes			
*Slc1a2* NM_001035233.1	CCAGTGCTGGAACTTTGCCT TAAAGGGCTGTACCATCCAT FAM-AGCGTGTGACCAGATTCGTCCTCCCA-BHQ1	200 150	[133] [45]
*Slc1a3* NM_019225.2	GCGCTGTCATTGTGGGTACA CAGAAGCTCCCCAGGAAAGG Cy5-CCTTGGATTTGCCCTCCGACCGT-BHQ3	200 100	[134]
*Slc1a1* NM_013032.3	CCTGCATCCCTCATCCCAC CTCCTACCACGATGCCCAGTA HEX-CCGCCGCGCTCCCCGATTCC-BHQ2	200 100	[134]
*Glul* NM_017073.4	CCTTTCGGCTGGCCTTCTAA GCTCCCACACCGCAGTAATA ROX-TGGCTTCCCTGGACCCCAAGGACC-BHQ2	200 150	[44]
Neuroinflammation genes			
*Nlrp3* NM_001191642	CAGACCCTCATGTTGCCTGT AGACCTCGGCAGAAGCTAGA FAM-CCAGACTGGTGAACTGCTGCCTCA-BHQ1	200 100	[134]
*Il1b* NM_031512	CACCTCTCAAGCAGAGCACAG GGGTTCCATGGTGAAGTCAAC FAM-TGTCCCGACCATTGCTGTTTCCTAG-BHQ1	400 200	[135]
*Il1rn* NM_022194.2	GGGGACCTTACAGTCACCTAAT GGTTAGTATCCCAGATTCTGAAGG ROX-AGTCAGCTGGCCACCCTGCTGGGA-BHQ2	400 100	[132]
Trophic factor genes			
*Bdnf* NM_001270630.1	CCATAAGGACGCGGACTTGTAC GAGGAGGCTCCAAAGGCACTT ROX-CTTCCCGGGTGATGCTCAGCAGT-BHQ2	400 200	[134]
*Fgf2* NM_019305.2	AGCGGCTCTACTGCAAGAAC TGGAGCTGTAGTTTGACGTGT R6G-AGACGGCCGCGTGGACGGCGTCCG-BHQ2	400 200	[134]
*Tgfb1* NM_021578.2	CTGCTGACCCCCACTGATAC AGCCCTGTATTCCGTCTCCT FAM-TGTCCGGCAGTGGCTGAACCA-BHQ1	200 100	[134]
Genes encoding markers of A1/A2 astrocyte states			
*Lcn2* NM_130741.1	AGCTACGATGTGCAAGTGGC CCCCTTGGTTCTTCCGTACA FAM-CGACACTGACTACGACCAGTTTGCCA-BHQ1	200 150	[134]
*Gbp2* NM_133624.2	AGTCAATGGGCCACGTCTAA AGTGGGTGATGGCCTTTTGT HEX-AGCAGTGGGTCTCTCCCCTGCA-BHQ2	200 100	[44]
*Ptx3* NM_001109536.2	AAACTTCGCCTCTCCAGCAA CATGGTGTGGGGTCCTCG HEX-TGCTCTCTGGTCTGCAGTGTTGGC- BHQ2	400 200	[44]
*S100a10* NM_031114.1	CATTTCACAGGTTTGCAGGGG GCACTGGTCCAGGTCTTTCA Cy5-AGGACCCTCTGGCTGTGGACA-BHQ3	200 250	[134]
Genes encoding markers of M1/M2 microglial states			
*Nos2* NM_012611.3	CAGAAGCAGAATGTGACCATCAT CGGAGGGACCAGCCAAATC ROX-ACCACCACACAGCCTCAGAGTCCTT-BHQ2	200 200	[136]
*Arg1* NM_017134.3	AGCTGGGAATTGGCAAAGTG AACTCAGGTGAATGGGCCTT HEX-TGGAAGAGACCTTCAGCTACCTGC-BHQ2	300 100	[137] [134]

**Table A2:** Antibodies used in the study.

Antibody	Clonality	Host Species	Dilution	Manufacturer
Primary antibodies				
Anti-GFAP (ab7260)	Polyclonal	Rabbit	1:20,000	Abcam (Cambridge, UK)
Anti-EAAT2 (ab205248)	Monoclonal	Rabbit	1:6000	Abcam (Cambridge, UK)
Anti-NMDAR1 (ab13345)	Polyclonal	Rabbit	1:1000	Abcam (Cambridge, UK)
Anti-GluN2a (ab169873)	Polyclonal	Rabbit	1:1000	Abcam (Cambridge, UK)
Anti-GluN2b (ab65783)	Polyclonal	Rabbit	1:1000	Abcam (Cambridge, UK)
Anti-GluA1 (ab109450)	Monoclonal	Rabbit	1:1000	Abcam (Cambridge, UK)
Anti-GluA2 (MAB397)	Monoclonal	Mouse	1:7500	Merck Millipore (Burlington, MA, USA)
Secondary antibodies				
Anti-rabbit IgG-HRP (31460)		Goat	1:20,000	Thermo Fisher Scientific (Rockford, IL, USA)
Anti-mouse IgG-HRP (31430)		Goat	1:25,000	Thermo Fisher Scientific (Rockford, IL, USA)

**Table A3:** Values obtained from statistical processing of data.

Short-Chain Fatty Acids (Figure 2)	
Propanoic acid	F_1,26_ (Interaction) = 0.528, *p* = 0.474 F_1,26_ (TLE) = 3.213, *p* = 0.085 F_1,26_ (Treatment) = 3.422, *p* = 0.076
2-Methylpropanoic acid	F_1,24_ (Interaction) = 2.016, *p* = 0.169 **F_1,24_ (TLE) = 7.721, *p* = 0.010** **F_1,24_ (Treatment) = 10.190, *p* = 0.004**
Butyric acid	F_1,25_ (Interaction) = 0.002, *p* = 0.16 **F_1,25_ (TLE) = 12.490, *p* = 0.002** **F_1,25_ (Treatment) = 20.910, *p* < 0.001**
3-Methylbutanoic acid	F_1,24_ (Interaction) = 3.174, *p* = 0.088 F_1,24_ (TLE) = 0.684, *p* = 0.416 F_1,24_ (Treatment) = 3.174, *p* = 0.088
Pentanoic acid	F_1,26_ (Interaction) = 2.911, *p* = 0.099 F_1,26_ (TLE) = 0.438, *p* = 0.514 **F_1,26_ (Treatment) = 5.741, *p* = 0.024**
4-Methylvaleric acid	F_1,26_ (Interaction) = 0.229, *p* = 0.636 F_1,26_ (TLE) = 0.959, *p* = 0.336 F_1,26_ (Treatment) = 2.636, *p* = 0.116
Hexanoic acid	F_1,26_ (Interaction) = 0.610, *p* = 0.442 F_1,26_ (TLE) = 1.088, *p* = 0.306 **F_1,26_ (Treatment) = 15.660, *p* < 0.001**
Heptanoic acid	F_1,26_ (Interaction) = 1.073, *p* = 0.310 F_1,26_ (TLE) = 2.775, *p* = 0.108 **F_1,26_ (Treatment) = 4.401, *p* = 0.046**
Open Field test (Figure 3)	
The total distance	F_2,36_ = 2.384, *p* = 0.107
Locomotion	F_2,35_ = 2.788, *p* = 0.075
Time in the center	H = 1.459, *p* = 0.482
Grooming, time	**F_2,33_ = 4.389, *p* = 0.02**
Climbings	F_2,17,5_ = 2.853, *p* = 0.085
Number of holes	**H = 8.377, *p* = 0.015**
The total time of hole inspections	**H = 11.79, *p* = 0.003**
Time of one hole investigation	**F_2,34_ = 4.343, *p* = 0.021**
RT-qPCR	
Ionotropic glutamate receptor subunit gene expression (Figure 4)	
*Grin1*	DH: **F_(2,16)_ = 9.239, *p* = 0.002** TC: **F_(2,17)_ = 7.155, *p* = 0.006**
*Grin2a*	DH: **F_(2,8.676)_ = 4.410, *p* = 0.048** TC: **F_(2,17)_ = 4.370, *p* = 0.029**
*Grin2b*	DH: **F_(2,17)_ = 6.726, *p* = 0.007** TC: F_(2,17)_ = 2.306, *p* = 0.130
*Gria1*	DH: **F_(2,9.257)_ = 15.942, *p* = 0.001** TC: **F_(2,8.050)_ = 19.612, *p* = 0.001**
*Gria2*	DH: **F_(2,16)_ = 9.789, *p* = 0.002** TC: **F_(2,17)_ = 7.952, *p* = 0.004**
Glutamate-glutamine cycle gene expression (Figure 6a)	
*Slc1a3*	DH: **F_(2,17)_ = 11.021, *p* = 0.001** TC: F_(2,17)_ = 1.760, *p* = 0.202
*Slc1a2*	DH: **F_(2,17)_ = 4.280, *p* = 0.031** TC: F_(2,18)_ = 1.187, *p* = 0.329
*Slc1a1*	DH: **F_(2,16)_ = 11.268, *p* = 0.001** TC: F_(2,18)_ = 0.841, *p* = 0.448
*Glul*	DH: **F_(2,17)_ = 5.958, *p* = 0.011** TC: F_(2,17)_ = 0.709, *p* = 0.506
Astrocyte and microglial marker gene expression (Figure 7a)	
*Gfap*	DH: **F_(2,8.883)_ = 118.217, *p* < 0.001** TC: **F_(2,18)_ = 96.363, *p* < 0.001**
*Aif1*	DH: **F_(2,17)_ = 15.952, *p* < 0.001** TC: **F_(2,18)_ = 6.595, *p* = 0.007**
*S100b*	DH: **F_(2,7.691)_ = 8.731, *p* = 0.010** TC: F_(2,18)_ = 0.928, *p* = 0.413
Gene expression of markers of A1/A2 astrocyte states (Figure 8)	
*Lcn2*	DH: **F_(2,18)_ = 15.239, *p* < 0.001** TC: **F_(2,9.810)_ = 13.625, *p* = 0.001**
*S100a10*	DH: **F_(2,18)_ = 12.052, *p* < 0.001** TC: **F_(2,18)_ = 10.486, *p* = 0.001**
*Gbp2*	DH: F_(2,18)_ = 1.096, *p* = 0.355 TC: F_(2,17)_ = 2.561, *p* = 0.107
*Ptx3*	DH: **F_(2,8.231)_ = 7.140, *p* = 0.016** TC: **F_(2,15)_ = 13.980, *p* < 0.001**
Gene expression of markers of M1/M2 microglial states (Figure 9)	
*Nos2*	DH: F_(2,16)_ = 0.228, *p* = 0.798 TC: **F_(2,13)_ = 6.170, *p* = 0.013**
*Arg1*	DH: **F_(2,18)_ = 6.536, *p* = 0.007** TC: **F_(2,15)_ = 7.295, *p* = 0.006**
Gene expression of neuroinflammation factors (Figure 10)	
*Nlrp3*	DH: **F_(2,18)_ = 17.826, *p* < 0.001** TC: **F_(2,16)_ = 7.153, *p* = 0.006**
*Il1b*	DH: **F_(2,17)_ = 3.675, *p* = 0.047** TC: F_(2,17)_ = 0.174, *p* = 0.842
*Il1rn*	DH: **F_(2,18)_ = 11.648, *p* = 0.001** TC: **F_(2,16)_ = 5.820, *p* = 0.013**
Trophic factor gene expression (Figure 11)	
*Bdnf*	DH: F_(2,8.138)_ = 0.473, *p* = 0.639 TC: **F_(2,9.843)_ = 21.840, *p* < 0.001**
*Fgf2*	DH: F_(2,18)_ = 3.270, *p* = 0.061 TC: F_(2,15)_ = 2.501, *p* = 0.115
*Tgfb1*	DH: **F_(2,18)_ = 23.978, *p* < 0.001** TC: **F_(2,11.162)_ = 22.629, *p* < 0.001**
Western blot (Figure 5, Figure 6b and Figure 7b)	
GluN1	F_2,15_ = 0.054, *p* = 0.948
GluN2a	**F_2,15_ = 3.917, *p* = 0.043**
GluN2b	F_2,15_ = 1.837, *p* = 0.193
GluA1	F_2,15_ = 0.996, *p* = 0.392
GluA2	F_2,14_ = 0.262, *p* = 0.773
GFAP	**F_2,15_ = 18.620, *p* < 0.001**
EAAT2	F_2,14_ = 2.526, *p* = 0.116
